# Functionalized Nanogap for DNA Read‐Out: Nucleotide Rotation and Current‐Voltage Curves

**DOI:** 10.1002/cphc.202000391

**Published:** 2020-08-20

**Authors:** Frank C. Maier, Maria Fyta

**Affiliations:** ^1^ Institute for Computational Physics Universität Stuttgart Allmandring 3 70569 Stuttgart Germany

**Keywords:** biosensors, density functional calculations, DNA, electron transport, molecular electronics, molecular recognition

## Abstract

Functionalized nanogaps embedded in nanopores show a strong potential for enhancing the detection of biomolecules, their length, type, and sequence. This detection is strongly dependent on the features of the target biomolecules, as well as the characteristics of the sensing device. In this work, through quantum‐mechanical calculations, we elaborate on representative such aspects for the specific case of DNA detection and read‐out. These aspects include the influence of single DNA nucleotide rotation within the nanogap and the current‐voltage (I‐V) characteristics of the nanogap. The results unveil a distinct variation in the electronic current across the functionalized device for the four natural DNA nucleotides with the applied voltage. These also underline the asymmetric response of the rotating nucleotides on this applied voltage and the respective variation in the rectification ratio of the device. The electronic tunneling current across the nanogap can be further enhanced through the proper choice of an applied bias voltage. We were able to correlate the enhancement of this current to the nucleotide rotation dynamics and a shift of the electronic transmission peaks towards the Fermi level. This nucleotide specific shift further reveals the sensitivity of the device in reading‐out the identity of the DNA nucleotides for all different configurations in the nanogap. We underline the important information that can be obtained from both the I‐V curves and the rectification characteristics of the nanogap device in view of accurately reading‐out the DNA information. We show that tuning the applied bias can enhance this detection and discuss the implications in view of novel functionalized nanopore sequencers.

## Graphical abstract

**An applied voltage bias** across a nanogap opened by diamondoid‐functionalized electrodes can be used to detect the identity of DNA nucleotides. For the detection, the different rotations of the DNA inside the nanogap control the exact value of the electronic current used for the detection. The applied bias leads to distinct IV curves and a nucleotide‐specific rectification of the nanogap device showing the high potential for DNA read‐out.

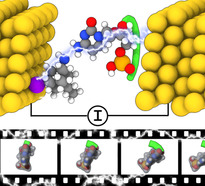

## 1 Introduction

Biosensors can detect the type of biomolecules, such as DNA, RNA, and proteins. DNA is a linear polymer that contains the genetic information of all known living organisms^1^ saved in four monomers, the nucleotides. Each of these is made of a nucleobase linked to a sugar phosphate backbone forming sequences of complementary nucleotides held by hydrogen bonds. These sequences can be read‐out by a class of novel next generation label‐free sequencing platforms involving nanopores. These are nanometer‐sized holes in materials that electrophoretically translocate and detect DNA.^2^, ^3^, ^4^ In order to read‐out the identity of DNA or any biomolecule that is threading the nanopore, longitudinal ionic^5^ and/or transverse electronic current^6^, ^7^, ^8^ signals are being measured. For the latter, metallic electrodes are embedded into the nanopore, across which a voltage difference can generate the electronic current expected to identify the DNA nucleotides passing through the nanopore. A strong enhancement of these electronic current signals leading to a low signal‐to‐noise ratio^9^ is expected when the embedded electrodes are functionalized.^10^ The functionalization is typically being made using molecules of the size of the nucleotides that promote a nucleotide specific interaction with the latter through hydrogen bonds.^11^, ^12^, ^13^

Although, the first bio‐based nanopore device has already been commercialized^14^ and many other materials^15^, ^16^, ^17^, ^18^, ^19^ and setups^8^, ^20^ are being investigated, open issues still remain.^21^ These are often related to a lower – than desired – signal‐to‐noise ratio, a too fast translocation speed, the high flexibility and very rich dynamics of the translocating DNA, the noise arising from the fluidic environment, etc. These factors hinder an error‐free, rapid, and low‐cost DNA sequencing through solid‐state nanopores. In order to enhance the signal‐to‐noise ratio, we have previously proposed the use of a tiny modified nanodiamond particle to functionalize the gold electrodes in a nanopore.^22^ This small particle is the smallest of a family known as diamondoids, which are nanoscale diamond‐like carbon nanocages terminated by hydrogen atoms.^23^ These have tunable optoelectronic properties, are thermodynamically very stable, vary in size and can be selectively modified.^24^ Derivatives of diamondoids,^25^ can be double modified in order to graft on the gold surface on one side, while on the other side these offer donor/acceptor sites for hydrogen binding to DNA nucleotides.^26^ We have already shown that such a device shows a high DNA selectivity also in the presence of a water solvent.^27^

Along these lines, we aim here to assess the influence of the DNA dynamics in such a functionalized nanogap and on the DNA detection. More importantly, here we focus on the nanogap device characteristics and discuss pathways for enhancing the differences among DNA nucleotides, that is improve read‐out, through the application of a bias voltage across the device. Accordingly, in this article we begin with an outline of the methodology used, analyze the main results on the influence of the DNA nucleotides and the applied bias on the electronic transmission of the nanogap device, and in the end discuss the conclusions and the relevance to novel DNA sequencers.

## 2 Methodology

**Figure 1:**
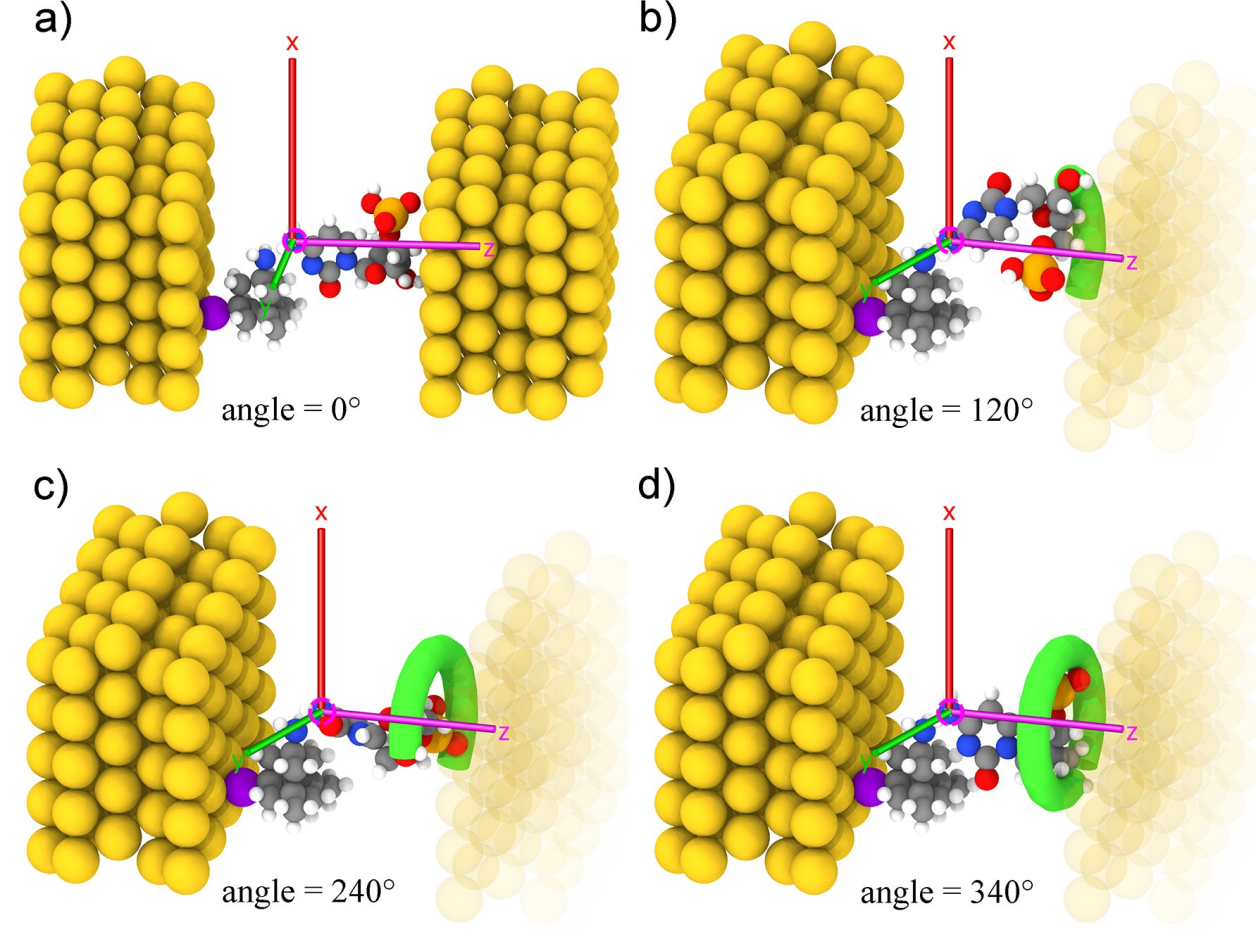
The setup of the functionalized device, with the memantine molecule grafting on the left electrode. The nucleotide (here cytosine) is placed close to the right electrode. The transport direction is from the left to the right electrode. The gold, sulfur, hydrogen, carbon, nitrogen, oxygen, phosphorus atoms are colored yellow, purple, white, gray, blue, red, and orange respectively. Panel a) shows the initial setup, with both the transport and rotation axis along the z‐axis. This setup corresponds to no rotation. The highlighted nitrogen atom is the rotation center on which the axes are placed and shown. In panel b), the nucleotide is rotated by 120, in c) by 240 and in d) by 340. In these panels, the right electrode is dimmed to emphasize the rotation.

The device we focus on is made of electrodes functionalized on the left side with an amine‐modified diamondoid derivative^28^ known as memantine (’mem’). Memantine is bio‐compatible and a drug used in Alzheimer's disease.^29^ Mem is anchored on the gold surface through a thiol group as shown in Figure 1. We place a single DNA nucleotide within the electrode gap. The semi‐infinite (in two directions) (111) gold electrodes are each represented by 5×5 gold atoms in 5 layers, which have a gap of 18 Å wide. The electrode are initially optimized, but are kept fixed during the geometry optimization of the molecules. The size of the supercell is 14.8×14.8×39.8 Å. The nanogap can accommodate the two molecules, nucleotide and diamondoid, which form hydrogen bonds and allow an electronic current to tunnel from the left to the right electrode. The molecules separately placed within the nanogap are the four DNA nucleotides, 2’‐deoxyadenosine 5’‐monophosphate (A), 2’‐deoxythymidine 5’‐monophosphate (T), 2’‐deoxycytosine 5’‐monophosphate (C), 2’‐deoxyguanosine 5’‐monophosphate (G). Mem and one nucleotide fill the gap between both electrodes in such a way, that the phosphate group of the nucleotide is oriented close to the right electrode. The initial placement (Figure 1(a)) assures that the amine group of the memantine forms hydrogen bonds to the nucleotide.

We perform quantum mechanical calculations based on the density functional theory (DFT)^30^, ^31^ as implemented in the code SIESTA.^32^ A single‐zeta plus and a double‐zeta plus polarization basis set are used for the gold electrodes and the atoms of the molecules, respectively. The Perdew‐Burke‐Ernzerhof (PBE‐GGA) functional^33^ was used as the exchange‐correlation functional and the pseudopotentials are of the norm‐conserving Troullier‐Martins type.^34^ The energy shift and the real space mesh cutoff are 0.01 Ry and 200 Ry, respectively. During the structural relaxation, the integration over k‐space is performed with 4×4×1 k‐points. The electronic and transport properties are calculated using a k‐space of 5×5×1 k‐points. The tolerance for the atomic forces was set to 1.1 eV/Å. All calculations are performed at the Γ‐point. As a starting point in this work, we use our previously optimized geometries for the functionalized nanogap.^28^

For the electronic transport calculations, the non‐equilibrium Green's functions (NEGF) formalism combined with DFT was employed as implemented in the code Transiesta.^35^ The tight‐binding transport calculations were done with TBTrans and a fine k‐point sampling of 10×10×1. The functionalized nanogap device is made up of left and right electrodes and the scattering region including the diamondoid and the nucleotide, as depicted in Figure 1. The left and right electrodes are the source and drain of electrons, respectively. In order to numerically solve the large tridiagonal matrix eigenvalue problem Green's functions are considered. For these, the electrode contribution of the left (right) electrode is given through the respective self‐energies and the overlap and Hamiltonian matrices of the scattering region are also taken into account. The energy‐resolved electronic transmission, *T* (*E*), can be obtained from the calculations and represents the probability of an electron injected from the left electrode to reach the right electrode. Zero‐bias calculations are performed with the chemical potential of both electrodes being at the same level. Under this condition, the electrical current is zero by definition and the transmission calculations predict the conductance of the device in the limit of small bias voltages. In order to obtain the current‐voltage characteristics, we perform bias dependent self consistent calculations. The current, (*I*), through the scattering region at an applied bias voltage, (*V*), is calculated through the integration of the transmission spectrum using the Landauer‐Büttiker formula^36^ through

(1) $$I\left(V\right)=\frac{2e}{h}\int_{-\mu_{R}}^{\mu_{L}}T\left(E,V\right)\left[f_{L}\left(E-\mu_{L}\right)-f_{R}\left(E-\mu_{R}\right)\right]dE,$$

with *T* (*E, V*) the bias dependent transmission probability of an electron with energy E. The functions *f_L_*(*E–μ_L_*), *fR*(*E–μ_R_*) are the Fermi‐Dirac distributions for the left and right electrode, respectively. The electrochemical potential for the left electrode and the right electrode is *μ_L_*=*E_F_*+*V/*2 and *μ_R_*=*E_F_* –*V/*2, respectively. The Fermi energy *E_F_* is shifted to zero. The bias voltage we have applied ranges from −1 V to +1 V in steps of 0.1 V. Note, that no solvent is present and a bias of ±1 V cannot dissociate water in our model. Further details on the theory and its numerical implementation can be found elsewhere.^22^, ^37^, ^38^

This methodology allows us to focus on two aspects: (i) the dynamics of the nucleotide in the nanogap, and (ii) the application of a bias voltage across the device. For the former, we concentrate solely on rotations of the nucleotide within the pore. These are representative of the rich dynamics effects arising from the nucleotide in the pore. It is not possible to theoretically account for all dynamics that influence a real nanopore setup. By assessing the influence of the two aspects above, we aim to make another step into understanding how a functionalized nanogap device can be selectively tuned based on the microstructural characteristics of the target molecules. These can further be used to evaluate the biosensitivity of such a device. For the investigation of DNA dynamics effects, the nucleotide is rotated at increments of 20 with respect to the diamondoid and along the axis connecting both electrodes as can be seen from the snapshots in Figure 1. At each rotation, both the nucleotide and the hydrogen atoms of the diamondoid are relaxed.

## 3 Results and Discussion

We begin the discussion of the results with the device characteristics. With this, we refer to the electronic current features across the memantine‐functionalized nanogap, in which one of the four natural DNA nucleotides is being placed. This electronic current, as well as pathways to enhance it, are essential for an error‐free DNA detection.

### 3.1 I–V Characteristics

**Figure 2:**
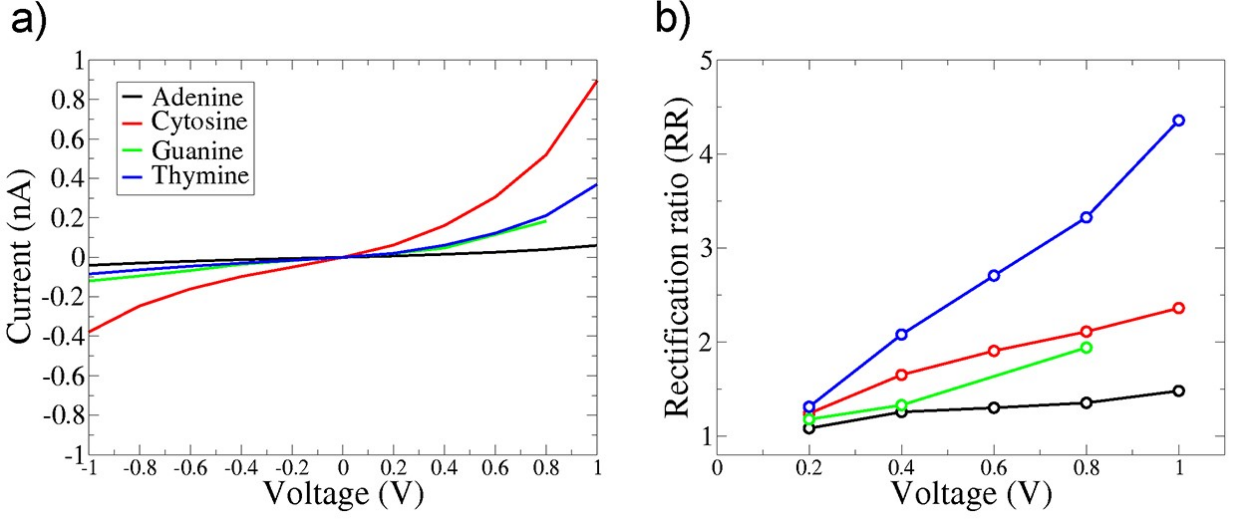
(a) The I‐V characteristics of the nanogap, for all nucleotides, for one specific configuration referring to a rotation of zero degrees, as denoted by the legend. The applied bias voltage is varied in the range [−1,+1] Volts. (b) The respective rectification ratio for the nanogap and all nucleotides.

As a first indication of the nanogap characteristics, we focus on the initial configuration in the nanogap, namely no rotation of the nucleotide. This configuration corresponds to the optimum (strongest) hydrogen bonding of the diamondoid and the nucleotide. We calculate the electronic current across the nanogap device with respect to the applied bias voltage across the functionalized electrodes. The respective I‐V characteristics for all four nucleotides are given in Figure 2. A first inspection of this figure reveals the very distinct I‐V curve for each nucleotide. Note, that we have omitted the calculation for the case of G at 1.0 V due to some numerical errors, since the trend in the IV curves is clear. Adenine shows an almost flat I‐V curve, cytosine corresponds to the highest current tunneling through the device, while thymine and guanine are in between and are closer to the adenine case. In all cases, though, the I‐V curves are not symmetric and show a rectification, which is stronger for cytosine. In order to visualize this, we include the rectification ratio (RR) in Figure 2(b). This is defined as *RR*(*V*)=*|I*(*V*)*/I*(*‐V*)*|*, where *I* and *V* are the current and applied bias voltage, respectively. The rectification is probably also related to the asymmetry of the hydrogen bonded complex of memantine and the nucleotide. Accordingly, a higher voltage enhances the differences of the nucleotides and leads to more specific signals, thus a better read‐out of the nucleotide identity. Interestingly, the rectification ratio is higher for thymine than for cytosine, though the current for the latter is larger. We observe that the trends do not change for the larger nucleotides, the purines A and G. The changes in these trends refer to the pyrimidines, C and T. We can thus sort the nucleotides by their tunneling current, in the decreasing sequence: cytosine, thymine, guanine and adenine. As a note on the comparison among the nucleotide data, a small difference in the absolute current values can lead to larger differences in the (rectification) ratio of these differences, taking also into account the asymmetry of the curves for positive and negative bias. A larger asymmetry in the curve, i. e. much higher absolute current values for a positive bias, lead to a larger rectification ratio. This is the reason for the larger differences in the RR for T and G compared with the much smaller differences in the respective electronic currents. From a physical point of view, once can observe that the higher asymmetry in the IV curves occur for T and C, while A and G relate to a lower asymmetry. Note, that T and C are the smaller pyrimidines, while A and G, the larger purines. Accordingly, our results underline the correlation of the molecule size with the IV asymmetry: smaller molecules lead to a higher asymmetry, thus a higher RR than larger molecules. The origin of this difference can probably be associated with larger upward shifts (with respect to the electrode's Fermi level) of the HOMO levels with positive bias for the pyrimidines as compared to the purines.^39^, ^40^ Note, that the larger size of the purines is also related to a stronger interaction with the right electrode related to a corresponding broadening of the molecular energy levels (data not shown), thus a lower current.^41^

**Figure 3:**
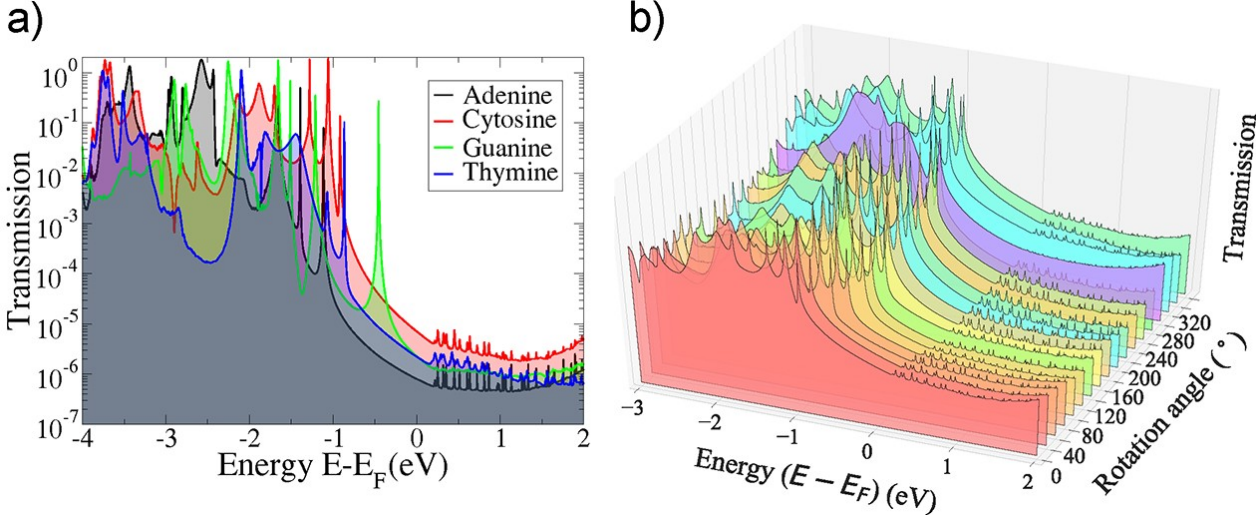
(a) The electronic transmission for no rotation with respect to the energy for all nucleotides as denoted by the legends. (b) The variation of the transmission as a function of both energy and rotation angle for cytosine. In both panels the energy is shown with respect to the Fermi (E_F_) level shifted to 0 eV.

In order to understand the I‐V characteristics, we turn to the electronic transmission for all nucleotides. This is obtained directly from our transport calculations. For the initial nucleotide positioning, no rotation in the pore (Figure 1(a)), the electronic transmission is depicted in Figure 3(a). Clearly distinct and nucleotide specific peaks are revealed denoting that the nucleotides can be distinguished from each other through these peaks. These are related to the specific hydrogen bonding of the diamondoid and the nucleotide.^26^ Our results show, that the transmission peaks for all rotations can be associated to peaks in the local electronic density of states (not shown) for all nucleotides. This strengthens our findings, that the electronic transmission is strongly nucleotide dependent no matter the rotation dynamics or the exact configuration in the nanogap. As the applied bias is being increased, larger parts of the electronic transmission function will contribute to the tunneling current. Note, that the tunneling current can be obtained by an integration of *T* (*E*) over the energy. This function will also change in response to the bias, as evident from the respective transmission curves (Figure 3(b)). Specifically, the increase of the bias voltage will result into more transmission peaks entering the integration window for the electronic current. Accordingly, more tunneling current is observed. This is consistent with the I‐V curves in Figure 2. Having in mind that electronic states close to the Fermi level promote the electronic transmission, we have calculated the contribution of the nucleotide and diamondoid electronic states to the transmission and density of states. For all nucleotides, these contribute to the lowest unoccupied molecular orbital (LUMO) states. In the case of the highest occupied molecular orbital (HOMO) level, C, G and T show sharp peaks, whereas the contribution of adenine is smaller than that of the diamondoid contribution. This justifies the lowest transmission, thus the less interesting I‐V curve for adenine.

### 3.2 Rotation Effects on Tunneling Current

The question that we further pose here is, how the nanogap device features change with the relative orientation of the functionalizing mem and the nucleotide. These changes are expected to influence the hydrogen bonding between the diamondoid and the nucleotide, thus the conductance across the nanogap.^42^ We provide the answer to the question above through the electronic transmission in Figure 3(b) for one of the nucleotides and the whole rotation range. It is evident from the figure that the sharp nucleotide specific transmission peaks remain throughout and are only being shifted to lower energies up to a rotation angle of about 240 degrees and to higher energies for larger rotation angles. The picture is qualitatively very similar for all four natural nucleotides. Comparing these results for all nucleotides leads to quantitative differences in the shift of the peaks and the respective peak energies.

**Figure 4:**
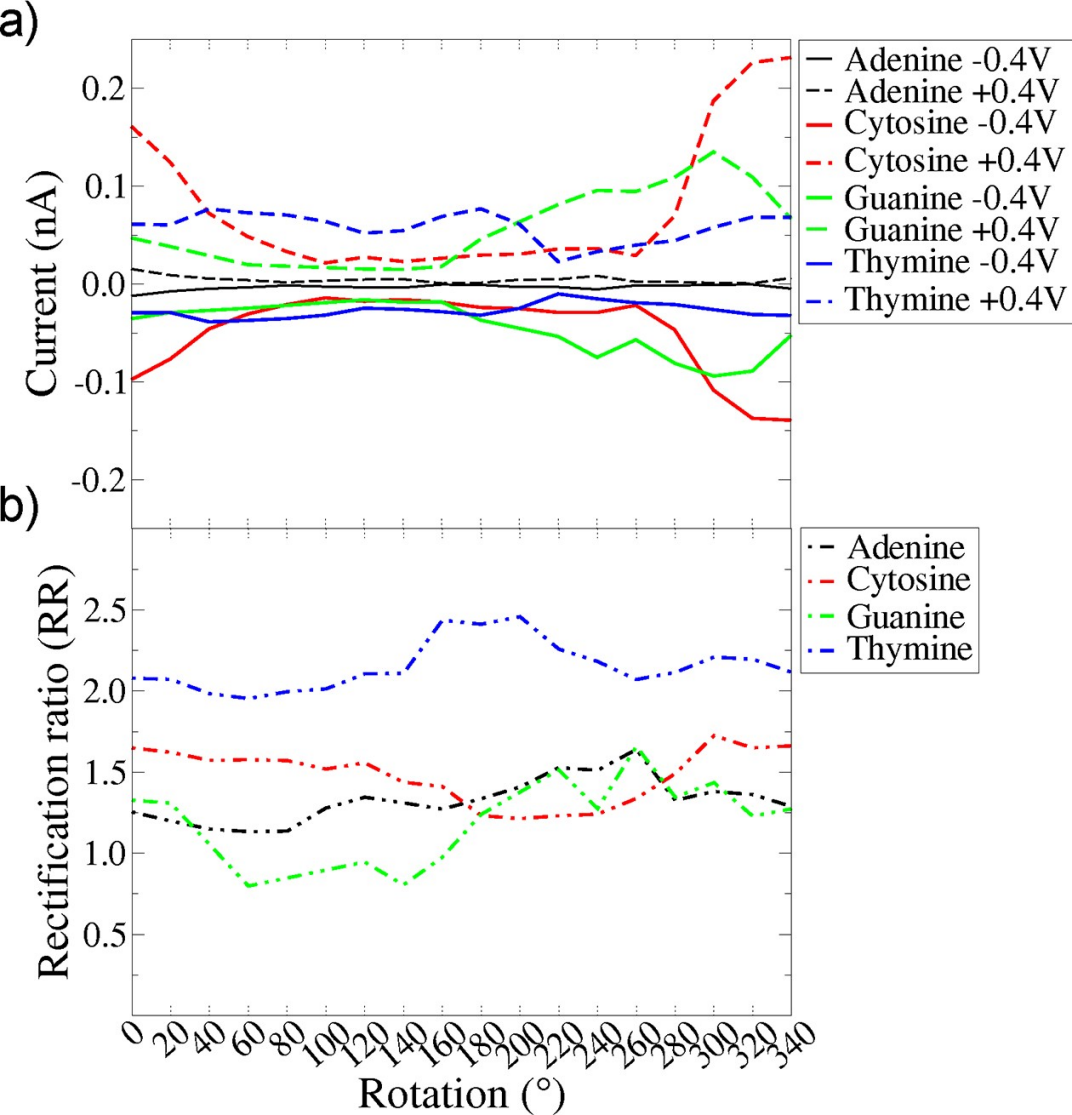
(a) The transverse electronic current across the nanogap with respect to the nucleotide rotation within the gap. Here, the data from Figure 2 are shown at the 0 point. The results are shown for all nucleotides performing a full rotation within the nanogap and two different values of the applied bias voltage, as denoted by the legends. (b) The respective rectification ratio related to the rotation dynamics.

In order to reveal the influence of the rotation dynamics on the tunneling current across the nanogap, we present the respective results in Figure 4(a), for two different values of the applied voltage and for all nucleotides. Note, that this figure shows the same quantities, but different results as Figure 2. All results shown in that figure refer to simulations with a fixed nucleotide geometry (referring to 0 deg.), but with a different applied bias. In Figure 4, the nucleotide is not fixed, but is being rotated along the transport direction. These rotations are performed at a fixed applied bias. At first, no specific trend can be observed in this figure. A comparison with Figure 2(a) reveals again that cytosine is associated with the highest absolute value of the current and adenine with the lowest. The current fluctuations evident in the cytosine case are larger than for the other cases, while the fluctuations for adenine are significantly smaller. Interestingly, the trends in the electronic current *|I_C_ |*<*|I_G_|*<*|I_T_ |*<*|I_A_|* (with *|I_X_ |* the absolute value of the electronic current associated with nucleotide *X*) do not hold throughout the rotation range. For intermediate rotations between 50–200, *|I_T_ |* is higher than *|I_C_ |*. The latter shows a sudden decrease close to 90 and rises again for larger rotation angles. This holds for both positive and negative values of the voltages shown in the figure. On the other hand, for higher rotations in the range 150–300, *|I_G_|* increases for both a positive and a negative bias voltage. Based on the analysis of the electronic transmission with the rotation as in Figure 3(b) for all nucleotides, we observe that these enhancements of the electronic currents for certain rotations correlate to the shift of the transmission peaks closer to the Fermi level. At a given rotation angle, these shifts are larger for the nucleotides associated with higher tunneling currents.

As a final result, we turn to Figure 4(b) and the rectification ratio as a function of the rotation dynamics. The observed trends vary with the rotation angle. Overall, thymine shows a larger rectification ratio than the other three nucleotides, consistent with the picture from Figure 2(b). This is followed by C and then by the purines, A and G. For a rotation angle larger than 200, the two purines show a similar rectification ratio, though these are related to a different current as seen in panel (b) of the same figure. It can be inferred from this that for such rotations it is mainly the similar size of the purines, which controls the rectification ratio and not their chemical specificity. This can more or less be observed also for the smaller pyrimidines, T and C. For these, the smallest thymine corresponds to a much larger rectification ratio. Overall, the different rotation dynamics keep the distinct features of the nucleotides in both the electronic tunneling current and the rectification ratio features. In view of detecting DNA sequences, both could be used with the former being mostly connected with the chemical details of the nucleotides and the latter mostly related to their size.

## 4 Conclusions

In this work, we have focused on two crucial aspects that are involved in nanopore detection using functionalized electrodes. These are the DNA dynamics represented by simple rotations of nucleotides within the nanogap and the control of the biosensitivity of the nanogap device through an applied bias voltage. We have performed quantum‐mechanical calculations to assess these effects and presented the trends observed with respect to the nucleotide identities. These differences are assessed with respect to the electronic transmission across the device and its electronic current characteristics. The I‐V curves for all nucleotides revealed rectification effects. For no rotation in the nanogap, cytosine was associated to the higher current followed by guanine, thymine, and adenine. This hierarchy did change when the rotation dynamics of the nucleotides was accounted for. We have observed that a higher current is correlated to transmission peaks closer to the Fermi level. This could be confirmed for both a positive and a negative applied bias voltage. Overall, our results unveil a distinct variation in the electronic current across the functionalized device for the four natural DNA nucleotides and strongly correlate to the dynamics of the molecular rotation, the shift of the electronic transmission peaks, the applied bias voltage, and the enhancement of the electronic tunneling current under certain conditions.

Through these results, we provide a proof‐of‐principles study on the characteristics of a functionalized nanogap that can be embedded into a nanopore. For this, we have neglected many other important aspects, such as more rich dynamics, dynamics of the functionalizing molecule as well, the influence of a fluidic environment or the presence of ions. Including all these effects is not an easy task and requires a step‐wise investigation. In any case, our work is one of the initial steps and can set a strong basis for further studies. Most importantly, it has revealed pathways to enhance the electronic current of the nucleotides through a tuning of the applied bias voltage across the nanogap. This enhancement has the potential to improve the biosensitivity of the device. We have proposed the use of both the I‐V curves and the rectification effects of the nanogap device as means for more accurately reading‐out the DNA information. Along these lines, this study is highly relevant to the design of novel nanopore sensors that can detect DNA sequences with a very high efficiency.

## Conflict of interest

The authors declare no conflict of interest.

## Supporting information

As a service to our authors and readers, this journal provides supporting information supplied by the authors. Such materials are peer reviewed and may be re‐organized for online delivery, but are not copy‐edited or typeset. Technical support issues arising from supporting information (other than missing files) should be addressed to the authors.

Supplementary
