# Supplementary material for: Functionalized Nanogap for DNA Read‐Out: Nucleotide Rotation and Current‐Voltage Curves
Source: Chemphyschem. 2020 Aug 20;21(18):2068–74. doi: 10.1002/cphc.202000391 (PMC7540481; doi:10.1002/cphc.202000391)
Supplement: Supplementary file 1 — Supplementary [file CPHC-21-2068-s001.pdf]

Author Contributions

M.F. Conceptualization:Equal; Supervision:Lead; Writing - Original Draft:Lead; Writing - Review & Editing:Lead
